# Clinical, physiologic, and radiographic factors contributing to development of hypoxemia in moderate to severe COPD: a cohort study

**DOI:** 10.1186/s12890-016-0331-0

**Published:** 2016-12-01

**Authors:** J. Michael Wells, Raul San Jose Estepar, Merry-Lynn N. McDonald, Surya P. Bhatt, Alejandro A. Diaz, William C. Bailey, Francine L. Jacobson, Mark T. Dransfield, George R. Washko, Barry J. Make, Richard Casaburi, Edwin J. R. van Beek, Eric A. Hoffman, Frank C. Sciurba, James D. Crapo, Edwin K. Silverman, Craig P. Hersh, the COPDGene Investigators

**Affiliations:** 1 Division of Pulmonary, Allergy, and Critical Care Medicine, University of Alabama Birmingham, Birmingham, AL USA; 2 Lung Health Center University of Alabama Birmingham, Birmingham, AL USA; 3 Birmingham VA Medical Center, Birmingham, AL USA; 4 Department of Radiology, Brigham and Women’s Hospital, Boston, MA USA; 5 Channing Division of Network Medicine, Brigham and Women’s Hospital, Harvard Medical School, Boston, MA USA; 6 Division of Pulmonary Medicine, Brigham and Women’s Hospital, Boston, MA USA; 7 Division of Pulmonary, Critical Care, and Sleep Medicine, National Jewish Health, Denver, CO USA; 8 Rehabilitation Clinical Trials Center, Los Angeles Biomedical Research Institute at Harbor UCLA Medical Center, Torrance, CA USA; 9 Department of Radiology, University of Edinburgh, Edinburgh, Scotland UK; 10 Department of Radiology, University of Iowa, Iowa City, IA USA; 11 Division of Pulmonary, Allergy, and Critical Care Medicine, University of Pittsburgh, Pittsburgh, PA USA; 12 1900 University Blvd, THT 422, Birmingham, AL 35294 USA

## Abstract

**Background:**

Hypoxemia is a major complication of COPD and is a strong predictor of mortality. We previously identified independent risk factors for the presence of resting hypoxemia in the COPDGene cohort. However, little is known about characteristics that predict onset of resting hypoxemia in patients who are normoxic at baseline. We hypothesized that a combination of clinical, physiologic, and radiographic characteristics would predict development of resting hypoxemia after 5-years of follow-up in participants with moderate to severe COPD

**Methods:**

We analyzed 678 participants with moderate-to-severe COPD recruited into the COPDGene cohort who completed baseline and 5-year follow-up visits and who were normoxic by pulse oximetry at baseline. Development of resting hypoxemia was defined as an oxygen saturation ≤88% on ambient air at rest during follow-up. Demographic and clinical characteristics, lung function, and radiographic indices were analyzed with logistic regression models to identify predictors of the development of hypoxemia.

**Results:**

Forty-six participants (7%) developed resting hypoxemia at follow-up. Enrollment at Denver (OR 8.30, 95%CI 3.05–22.6), lower baseline oxygen saturation (OR 0.70, 95%CI 0.58–0.85), self-reported heart failure (OR 6.92, 95%CI 1.56–30.6), pulmonary artery (PA) enlargement on computed tomography (OR 2.81, 95%CI 1.17–6.74), and prior severe COPD exacerbation (OR 3.31, 95%CI 1.38–7.90) were independently associated with development of resting hypoxemia. Participants who developed hypoxemia had greater decline in 6-min walk distance and greater 5-year decline in quality of life compared to those who remained normoxic at follow-up.

**Conclusions:**

Development of clinically significant hypoxemia over a 5-year span is associated with comorbid heart failure, PA enlargement and severe COPD exacerbation. Further studies are needed to determine if treatments targeting these factors can prevent new onset hypoxemia.

**Trial registration:**

COPDGene is registered at ClinicalTrials.gov: NCT00608764 (Registration Date: January 28, 2008)

**Electronic supplementary material:**

The online version of this article (doi:10.1186/s12890-016-0331-0) contains supplementary material, which is available to authorized users.

## Background

Hypoxemia is a major complication of chronic obstructive pulmonary disease (COPD) and is associated with increased mortality [1] and impaired exercise tolerance [2]. Hypoxemia contributes to other COPD-related complications including pulmonary vascular disease [3], skeletal muscle dysfunction [4], and polycythemia [5]. Resting hypoxemia has major implications related to morbidity, therapy, and prognosis, unlike exercise induced desaturation and nocturnal desaturation in COPD which have less well-defined prognostic significance [6–9]. The Centers for Medicare and Medicaid Services (CMS) defines the clinical threshold for hypoxemia in stable COPD as arterial oxygenation (PaO_2_) ≤55 mmHg or pulse oximetry (SpO_2_) ≤88% [10–12]. Treatment of resting hypoxemia with supplemental oxygen therapy is one of the few therapies for COPD that improves quality of life, exercise tolerance, and mortality [13–15]; thus, it is important to identify risk factors for its development.

Previously, in cross sectional analyses, we demonstrated that female sex, increased body mass index (BMI), lower forced expiratory volume in 1-s (FEV_1_), and high altitude (Denver, altitude 5280 ft) were independently associated with resting oxygen saturation of ≤88% on ambient air [16]. However, there is a paucity of information regarding the development and impact of new onset hypoxemia in COPD. Therefore, we hypothesized that a combination of clinical, physiologic, and radiographic characteristics would predict development of resting hypoxemia after 5-years of follow-up in participants with moderate to severe COPD. Further, we hypothesized that incident hypoxemia would be associated with negative impacts on quality of life, exercise performance, and lung function.

## Methods

### Subject selection

COPDGene is a prospective observational study conducted at 21 clinical centers across the U.S., including National Jewish Hospital in Denver [17]. In Phase 1, COPDGene recruited 10,192 non-Hispanic white and African-American current and former smokers (10 or more pack-years) 45 years old or older with and without COPD [17]. Participants are now returning for a Phase 2 visit approximately 5-years after their original visit. All study participants were at baseline health status, defined as no AECOPD within the previous 4 weeks, no surgery, acute myocardial infarction, or hospitalization for a cardiac problem within the previous 3 months, to standardize conditions between Phase 1 and Phase 2 visits. Our analysis used an interim dataset of the first 2000 Phase 2 participants. We included participants with moderate to very severe COPD defined by Global Initiative for Chronic Obstructive Lung Disease (GOLD) [18] spirometry grade 2-4 COPD at Phase 1, who did not have resting hypoxemia at baseline. COPDGene was approved by the Institutional Review Boards at participating institutions (Additional file 1: Table S1, online supplement).

### Oxygen saturation measurement

Oxygen saturation was measured by pulse oximetry on ambient air at both visits [17]. Pulse oximetry was obtained with the subject in the seated position and the value was recorded after the subject remained at rest in the seated position for at least 5 min. The pulse oximeter was placed on a finger without nail polish. The median value obtained while observing the monitor over a 1-min observation period was recorded. Pulse oximeters were calibrated on a regular basis on a schedule determined by the manufacturer’s recommendation and the local clinical center bioengineering department. Study coordinators were adequately trained in measurement in pulse oximetry. If participants used supplemental oxygen, oxygen was discontinued for 5 min prior to measurement of pulse oximetry. If the oxygen saturation fell below 82%, supplemental oxygen was restarted and 82% was recorded as the resting value. Hypoxemia was defined as an oxygen saturation ≤88% on ambient air [13, 14].

### Study procedures

Both visits included spirometry before and after 2 puffs of albuterol [19], 6 min walk testing (6MWT) [20], and the St. George’s Respiratory Questionnaire (SGRQ) [21], a measurement of quality of life (higher scores indicate worse quality of life) and the modified medical research council (MMRC) [22] dyspnea score. Comorbidities were defined by subject self-report. Coronary artery disease was defined as a positive response to history of coronary artery disease, angina, myocardial infarction, percutaneous coronary intervention, or coronary artery bypass graft surgery.

### Imaging

Participants underwent inspiratory and expiratory computed tomography (CT) scans of the chest at the baseline visit [17]. Emphysema was quantified by the percent of low attenuation areas (%LAA) < -950 HU on full inspiration [23] and gas trapping was quantified by %LAA < -856 HU at relaxed expiration [24] using Slicer software (www.slicer.org). The pulmonary artery (PA) and ascending aorta (A) diameters were measured as previously reported; a PA/A ratio >1 indicated PA enlargement [25, 26].

### Statistical analysis

Hypoxemia was defined by a resting O_2_ saturation ≤ 88% measured at the Phase 2 visit. Univariable logistic regression was used to define associations between Phase 1 variables and the development of hypoxemia at Phase 2. Variables associated at alpha < 0.10 on univariable analysis were entered into a multivariable backward logistic regression model to include all potential factors associated with hypoxemia at phase 2. Given the large percent of individuals recruited from the Denver site and the expected impact of living at altitude, we performed a sensitivity analysis using the multivariable logistic regression model on the subgroup of participants not recruited in Denver. T-tests were used to analyze differences in outcome measures (change in SGRQ, MMRC, 6MWT, and FEV_1_) between those who did and did not develop hypoxemia. Significance was defined as a *P* < 0.05. Analysis used SPSS for Windows Version 23 (IBM, USA).

## Results

### Subject characteristics

**Fig. 1:**
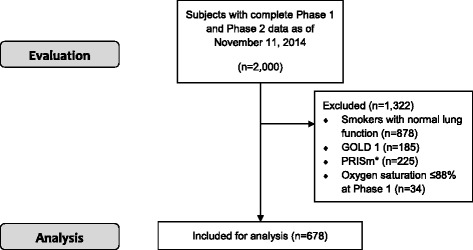
Patient Flow Diagram. Abbreviations: PRISm = Preserved Ratio, Impaired Spirometry

Of the 2000 participants who completed Phase 2 visits, *n* = 678 met inclusion criteria (Fig. 1). As seen in Table 1, participants were 63 ± 8 years old at Phase 1, 77% were non-Hispanic white, and 55% were male with a predicted FEV_1_ of 54 ± 16%. A total of 132 (19%) individuals were enrolled in Denver. At baseline, heart rate was 76 ± 12 beats/min and oxygen saturation was 95.5 ± 2.4%. Self-reported comorbid conditions included coronary artery disease (15%), congestive heart failure (3%), diabetes (11%), sleep apnea (20%), thromboembolic disease (4%), peripheral vascular disease (3%), and cerebrovascular disease (5%). On CT imaging, mean percent emphysema was 12.7 ± 11.7%, gas trapping was 38 ± 19%, and 19% of participants had a PA/A ratio >1.

**Table 1:** Baseline characteristics from Phase 1

Variable	*n* = 678
Age, years	63 ± 8
Non-Hispanic white race	525 (77%)
Male sex	375 (55%)
Enrollment at Denver clinical site	132 (19%)
BMI, kg/m^2^	28.2 ± 5.8
Baseline heart rate, beats/min	76 ± 12
Baseline oxygen saturation, %	95.5 ± 2.4
Current Smokers	248 (37%)
Pack Year History	52 ± 26
Coronary Artery Disease	102 (15%)
Congestive Heart Failure	20 (3%)
Hypertension	315 (47%)
Hyperlipidemia	277 (41%)
Asthma	178 (26%)
Sleep apnea	137 (20%)
Thromboembolic disease	26 (4%)
Peripheral vascular disease	23 (3%)
Cerebrovascular disease	36 (5%)
Gastroesophageal reflux disease	215 (32%)
FEV_1_, percent predicted	54 ± 16
FEV_1_/FVC	0.51 ± 0.12
GOLD 2	405 (59.7%)
GOLD 3	215 (31.7%)
GOLD 4	58 (8.6%)
Percent emphysema, -950 HU	12.7 ± 11.7
Percent gas trapping	38 ± 19
PA/A ratio	0.89 ± 0.13
PA/A ratio >1	127 (19%)
Severe AECOPD 1-year prior	101 (15%)
MMRC	1.8 ± 1.4
SGRQ, total	35 ± 21
6MWD, ft	1277 ± 361

Values represent mean ± SD or n (%)

*Definitions*: *FEV*_1_ forced expiratory volume in 1-s, *FVC* forced vital capacity, *GOLD* Global initiative for obstructive lung disease, *PA* pulmonary artery, *A* aorta, *AECOPD* acute exacerbation of COPD, *MMRC* modified medical research council, *SGRQ* St. George’s Respiratory Questionnaire, *6MWD* 6-min walk distance

### Change in oxygen saturation over 5-years

**Fig. 2:**
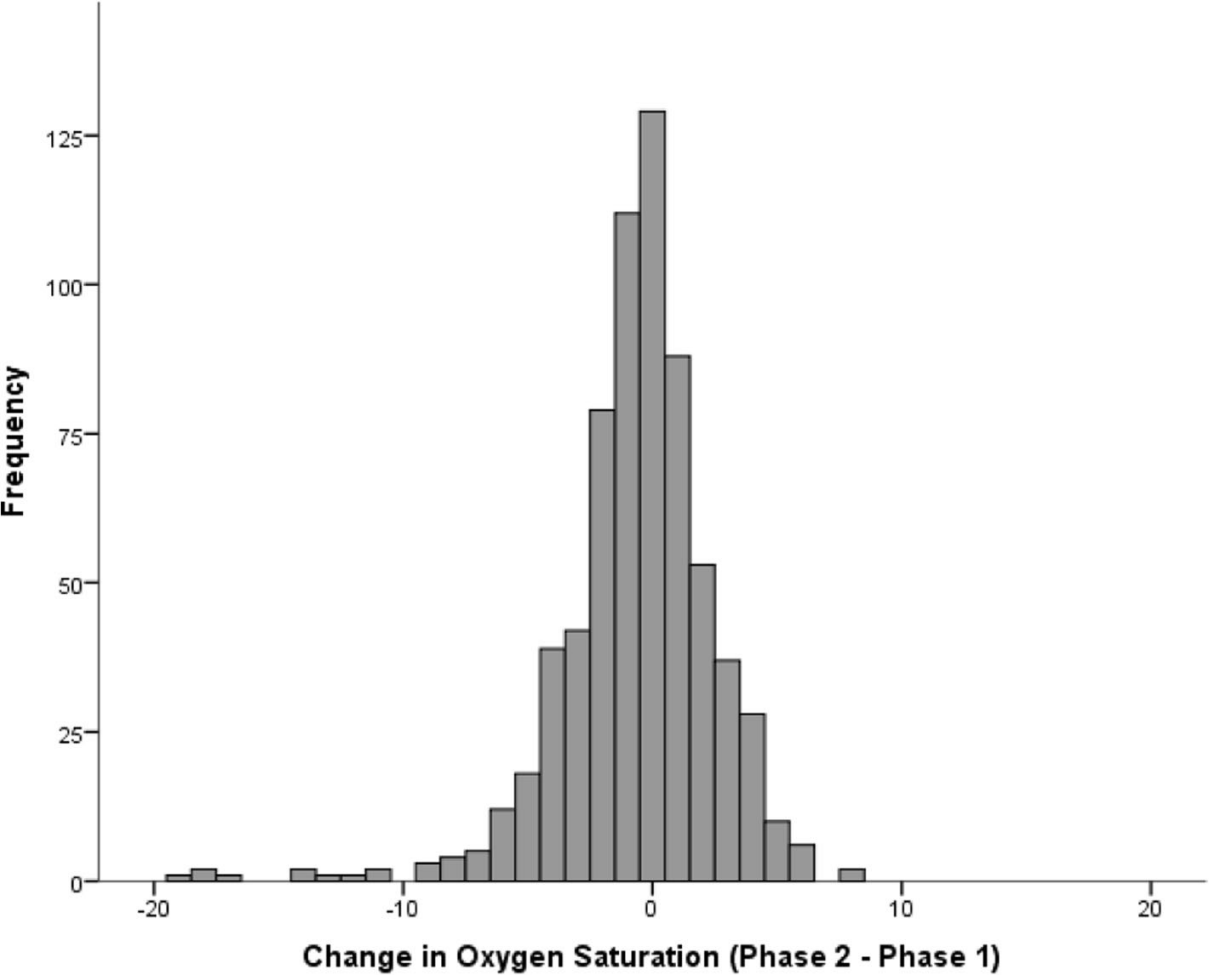
Distribution of change in oxygen saturation over a 5-year period among COPD cases without hypoxemia at baseline (*n* = 678)

The median follow-up time was 64 (range 51–78) months. As exhibited in Fig. 2, the change in resting oxygen saturation was normally distributed with a mean decrease of 1% over the 5-year period. Between Phase 1 and Phase 2 visits, 46 patients (7%) of patients developed hypoxemia at Phase 2.

### Predictors of the development of hypoxemia at rest

Development of a resting oxygen saturation ≤88% at Phase 2 was predicted by several baseline variables using univariable logistic regression (Table 2). In the multivariable model, enrollment at Denver, lower oxygen saturation at baseline, self-reported CHF, PA/A ratio >1, and having a severe AECOPD within 12-months prior to study entry were independently associated with the development of hypoxemia (Table 3). We observed a negative correlation between baseline CT percent emphysema and follow-up oxygen saturation (*R* = -0.23, *P* < 0.001). COPD cases who developed hypoxemia had higher baseline percent emphysema than those who did not develop hypoxemia (15.8 ± 10% versus 12.5 ± 10%, *P* = 0.015), though emphysema did not independently contribute to the regression model. Percent gas trapping on CT was not significantly elevated in cases that developed hypoxemia compared to those who did not (43.1 ± 13.7% versus 37.6 ± 19%, *P* = 0.057).

**Table 2:** Variables associated with the development hypoxemia at rest among COPD cases

Variable	OR	95% CI	*P*-value
Age, per 1 year increase	1.05	1.01–1.09	0.021
African American race	3.24	1.14–9.18	0.027
Male sex	1.41	0.76–2.62	0.28
Enrollment at Denver clinical site	12.2	6.26–23.6	<0.001
BMI, kg/m^2^	0.98	0.93–1.04	0.47
Baseline heart rate, beat/min	1.02	0.99–1.04	0.20
Baseline oxygen saturation, %	0.62	0.54–0.70	<0.001
Current Smokers	0.67	0.34–1.29	0.23
Pack Year History	1.01	1.00–1.02	0.05
Coronary artery disease	1.41	0.66–3.02	0.38
Congestive heart failure	5.02	1.74–14.5	0.003
Hypertension	0.59	0.32–1.11	0.10
Hyperlipidemia	1.23	0.68–2.25	0.49
Asthma	1.28	0.97–1.70	0.08
Sleep apnea	1.28	0.96–1.70	0.09
Thromboembolic disease	1.15	0.26–5.02	0.85
Peripheral Vascular Disease	1.32	0.30–5.81	0.71
Cerebrovascular Disease	1.26	0.37–4.29	0.71
Gastroesophageal reflux	1.72	0.94–3.16	0.08
FEV_1_, percent predicted	0.98	0.96–0.99	0.02
Percent emphysema	1.02	0.99–1.05	0.09
Percent gas trapping	1.02	0.99–1.03	0.09
PA/A ratio >1	2.02	1.04–3.92	0.038
Severe AECOPD within 12 months	2.98	1.56–5.67	0.001

Data represents univariable associations as determined by logistic regression analysis

*Abbreviations*: *OR* odds ratio, *CI* confidence interval, *BMI* body mass index, *FEV*_1_ forced expiratory volume in 1-s, *FVC* forced vital capacity, *PA*/*A* pulmonary artery to aorta ratio, *AECOPD* acute exacerbation of chronic obstructive pulmonary disease

**Table 3:** Independent predictors of incident resting hypoxemia in individuals with COPD

Variable	OR	95% CI	*P*-value
Enrollment at Denver clinical site	8.30	3.05–22.6	<0.001
Baseline oxygen saturation, %	0.70	0.58–0.85	<0.001
Congestive heart failure	6.92	1.56–30.6	0.01
PA/A ratio >1	2.81	1.17–6.74	0.02
Severe AECOPD within 12-months	3.31	1.38–7.90	0.007

Variables included in the backwards multivariate logistic regression model: age, race, sex, Denver, baseline oxygen saturation, pack-year history of tobacco use, congestive heart failure, hypertension, asthma, sleep apnea, gastroesophageal reflux disease, FEV_1_ percent predicted, percent emphysema, percent gas trapping, PA/A > 1, severe AECOPD within 12-months of visit (*R* ^2^ = 0.39, *P* < 0.001). Abbreviations: OR = odds ratio, CI = confidence interval, PA/A = pulmonary artery to aorta ratio, AECOPD = acute exacerbation of chronic obstructive pulmonary disease

We performed a sensitivity analysis by excluding the 132 participants (*n* = 32 who developed hypoxemia) enrolled in Denver. As demonstrated in Table 4 **,** lower baseline oxygen saturation, CHF, and a previous severe exacerbation remained independently associated with the development of hypoxemia, mirroring findings from the entire cohort.

**Table 4:** Multivariable logistic associations with the development of hypoxemia in subjects recruited from non-Denver sites

Variable	OR	95% CI	P-value
Baseline oxygen saturation, %	0.77	0.60–0.99	0.04
Congestive heart failure	7.22	1.66–31.3	0.008
Severe AECOPD within 12 months	4.26	1.33–13.6	0.015

This analysis is of the subgroup (*n* = 546) not recruited at Denver. Variables included in the backwards stepwise logistic regression model: age, race, sex, baseline oxygen saturation, congestive heart failure, FEV_1_ percent predicted, PA/A > 1, and severe AECOPD within 12-months of visit

*Abbreviations*: *OR* odds ratio, *CI* confidence interval, *BMI* body mass index, *FEV*_1_ forced expiratory volume in 1-s, *FVC* forced vital capacity, *PA*/*A* pulmonary artery to aorta ratio, *AECOPD* acute exacerbation of chronic obstructive pulmonary disease

### Associations between incident hypoxemia, quality of life, and 6-min walk distance

**Fig. 3:**
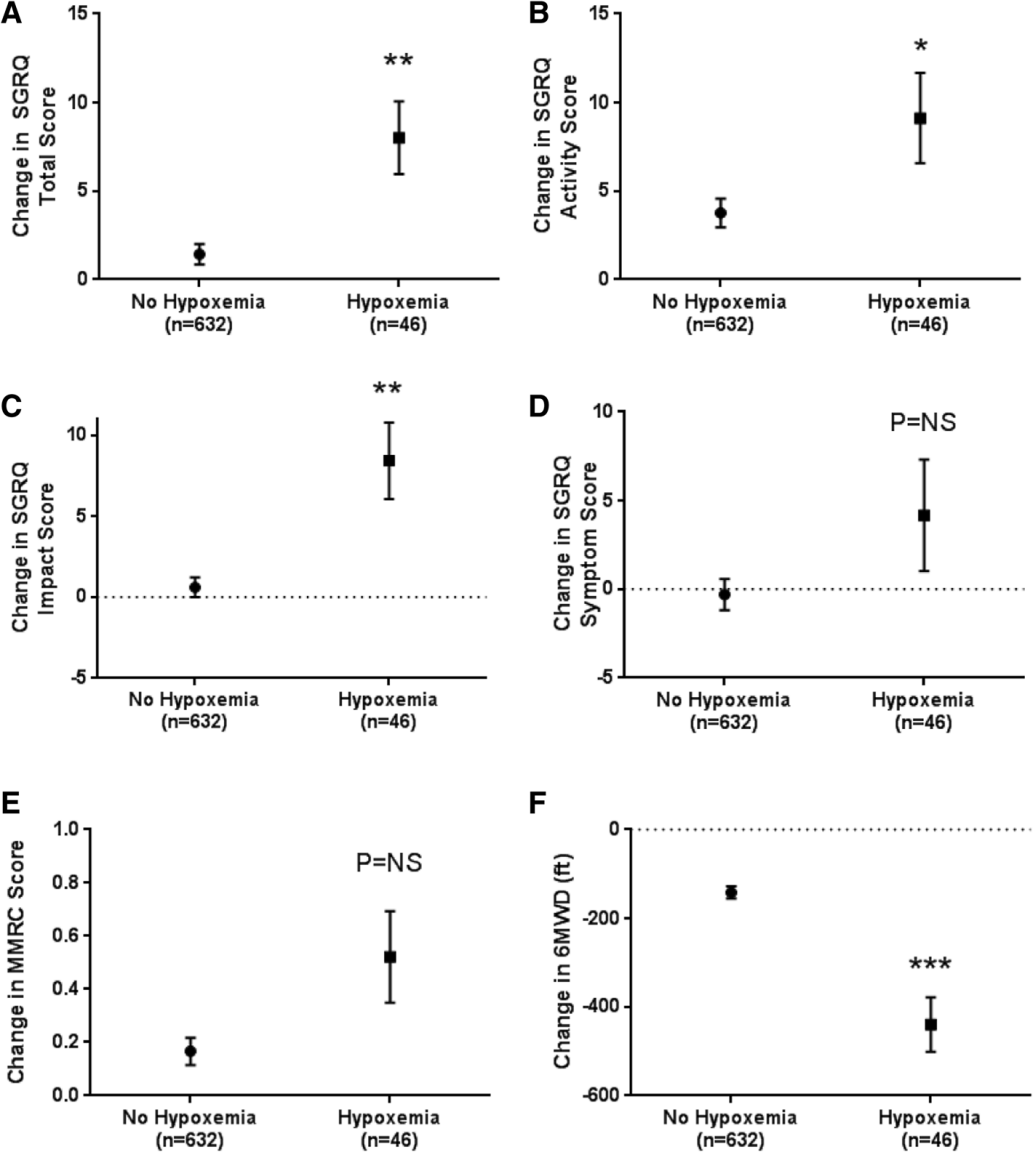
Development of hypoxemia is associated with worsened QOL and reduced exercise tolerance. Compared to normoxic participants at Phase 2, participants who developed hypoxemia had a greater rate of change in **a** SGRQ total score, **b** SGRQ activity score, **c** SGRQ impact score, but no difference of change in **d** SGRQ symptom score or breathlessness measured by **e** MMRC. Likewise, these participants have a greater decrease in **f** 6MWD change compared to participants who remained normoxic. Error bars represent standard error. SGRQ = St George’s Respiratory Questionnaire, 6MWD = 6-min walk distance. MMRC = modified medical research council score. *P < 0.05, **P < 0.01, ***P < 0.001

The development of hypoxemia adversely impacted quality of life, exercise tolerance, and dyspnea (Fig. 3). The change in total SGRQ (8.0 ± 2.1 versus 1.4 ± 0.6 points, *P* = 0.003), activity subscore (9.1 ± 2.5 versus 3.8 ± 0.8, *P* = 0.05), and impact subscore (8.5 ± 2.4 versus 0.6 ± 0.6, *P* = 0.002) were significantly higher in COPD cases who did vs. those who did not develop resting hypoxemia. The difference in change in SGRQ symptom score was not significant (4.2 ± 3.1 versus -0.3 ± 0.9, *P* = 0.18). COPD cases who developed hypoxemia had a trend towards increased dyspnea (change in MMRC 0.52 ± 0.17 versus 0.17 ± 0.05, *P* = 0.053). We observed a 3-fold greater decline in 6MWD (-440 ± 62 versus -141 ± 14 ft. [-134 ± 19 versus -43 ± 4 m], *P* < 0.001) in participants who developed hypoxemia.

## Discussion

We showed that there is substantial variability in the change in oxygen saturation over a 5-year period in non-hypoxemic participants with moderate to severe COPD. Hypoxemia develops in an appreciable proportion, and we identified multiple risk factors that are associated with incident hypoxemia at rest. Modifiable risk factors include living at high altitude and COPD exacerbations, and non-modifiable risk factors include baseline oxygen saturation, comorbid CHF, and pulmonary arterial enlargement on CT. Incident hypoxemia was associated with accelerated FEV_1_ decline, poorer quality of life, and shorter 6MWD.

Our results were consistent with prior analyses examining the longitudinal change in oxygen saturation and add to our understanding by adding imaging based metrics to clinical factors known to be associated with hypoxemia. In a population-based study of 2822 participants in Norway including 23.1% with COPD, Vold and colleagues reported that there was not an overall change in mean oxygen saturation over 6.3 years [27]. Only 25 participants (0.9%) in their study developed an oxygen saturation ≤92%, whereas 14% in our study developed an oxygen saturation ≤88%. Possible explanations for the differences between studies are the inclusion of participants enrolled at high altitude in COPDGene as well as genetic and racial variations. Despite these differences in study outcomes, Vold et al identified male sex, FEV_1_ ≤ 50% predicted, ≥10 pack-years smoking history, BMI ≥30 kg/m^2^, and C-reactive protein ≥5 mg/L as independent predictors of an oxygen saturation decline of ≥2%. In another 3-year study of 419 participants with GOLD 2 to 4 stage COPD, Saure and colleagues found no significant change in P_a_O_2_ over time, but 15% of the normoxic participants developed at least one episode of hypoxemia [28]. They did not identify any variables predictive of change in P_a_O_2_.

While the overall change in oxygen saturation was similar between our findings and prior publications, we found that hypoxemia develops in an appreciable portion (7%) of participants with moderate-to-severe COPD in our cohort. The factors associated with resting hypoxemia are related to lower resting oxygen saturation at baseline, episodic hospitalizations for AECOPD, chronic effects of high altitude, and cardiovascular comorbidities. The major contributor to impaired gas exchange in COPD is ventilation/perfusion mismatch due to airflow limitation [29] and emphysematous destruction of the capillary bed [30]. Gas exchange abnormalities present in stable COPD acutely worsen during AECOPD due to effects of inflammation, exaggerated V/Q mismatch, and increased oxygen consumption [31]. Our observations that low FEV_1_ and previous severe AECOPD contribute to oxygen saturation decline and incident hypoxemia are expected sequelae of these pathophysiologic processes. This leads one to posit the possibility that earlier and more aggressive treatment of underlying lung disease or preventing/minimizing inflammation following AECOPD may forestall the progression of hypoxemia.

Several factors associated with incident hypoxemia and oxygen saturation decline are related to cardiovascular and pulmonary vascular disease. We found that high altitude had the strongest association. It is well established that chronic residence at high altitude is associated with an increased mortality and higher incidence of *cor pulmonale* in COPD [32, 33]. While short-term studies have been performed evaluating the contribution of altitude on hypoxemia in COPD [34], our findings are the first to report long term changes in oxygen saturation in COPD patients living at high altitude. Given that living in Denver was such a strong independent predictor of the development of hypoxemia in COPD, this might suggest that patients with COPD should be advised to move to lower altitudes if these findings are recapitulated in additional studies.

Pulmonary arterial enlargement was the only chest CT metric that was associated with development of hypoxemia. We have previously shown that the PA/A ratio correlates with invasive hemodynamic measurements [35], early right ventricular dysfunction [25], and is predictive of severe AECOPD [26]. Pulmonary arterial enlargement likely represents a common measurable endpoint for the consequences of prolonged exposure to high altitude, ventricular failure, or idiopathic pulmonary hypertension. Although CT emphysema was higher in participants who developed hypoxemia and correlates with follow-up oxygen saturation, it did not independently contribute to oxygen saturation decline. These findings are in agreement with Dournes and colleagues’ recent work [36] suggesting that CT metrics of vascular and airway remodeling, but not emphysema, predict oxygen saturation and pulmonary hypertension in COPD. It is possible that other CT metrics not measured in COPDGene, including lung surface area [37] or small vessel volumes [25, 38] could also be related to the development of hypoxemia.

We also found a strong association between self-reported CHF and the development of hypoxemia. Patients with CHF, like those with COPD, depend on hypoxic vasoconstriction to maintain normal gas exchange [39]. While CHF is not commonly associated with hypoxemia in the absence of other contributing factors, heart failure in the context of *cor pulmonale* is related to gas exchange abnormalities and hypoxemia in COPD [40]. Consequences of concomitant chronic left ventricular impairment and COPD could be contributory to the development of hypoxemia [41] including residual gas exchange abnormalities stemming from episodic hypoxemia due to heart failure exacerbations [42, 43], intravascular shunting [44], or thromboembolic disease [45].

Incident resting hypoxemia had important implications. Poor quality of life, measured by the SGRQ, is associated with dyspnea, depression, and anxiety, and carries an increased risk for hospitalization and death [46, 47]. Hypoxemia also contributes to worsened SGRQ scores [48], though supplemental oxygen does not improve SGRQ scores in hypoxemic [49] and normoxic patients [50], highlighting the importance of addressing modifiable risk factors to prevent the decline in oxygen saturation. Small studies have shown that long-term supplemental oxygen therapy prevents exercise induced desaturation, improves walk distance, and improves dyspnea [8, 51] while others show limited benefit of therapy [52–54]. Clinical equipoise regarding treatment of mild-to-moderate hypoxemia in COPD may be addressed by the results of the upcoming long-term oxygen treatment trial (LOTT) [55].

Our study had several limitations. First, due to the observational nature, we were unable to assign causality between the risk factors identified and development of hypoxemia. Comorbidities were self-reported and may reflect over- or under-representation of comorbid conditions in this COPD cohort. Our study was also limited by the interim data analysis of the first 2000 Phase 2 participants; these results may not be reflective of the entire cohort. Additionally, we used pulse oximetry to assess oxygen saturation as arterial blood gases were not included in the COPDGene study protocol. While arterial blood gas analysis remains the gold standard for measuring oxygen concentration in COPD, pulse oximetry is widely used as a surrogate for evaluating hypoxemia in clinical practice [56, 57], increasing the generalizability of our findings. Finally, oxygen saturations were only measured on two occasions, limiting further conclusions about disease trajectory and raising the question about variability. When Saure and colleagues measured PaO_2_ at multiple intervals over a 3-year period, they found highly conserved values [28], suggesting that our method and findings are valid.

## Conclusions

Hypoxemia remains a critical development in the natural history of COPD. Our findings provide some insight into the complexities of the development and progression of hypoxemia in moderate to severe COPD and highlight the important contribution that high altitude and cardiovascular disease have on its incidence and may represent additional targets for future interventions.

## Additional file

Additional file 1: Table S1. IRB Approval and Protocol Numbers for COPDGene. (DOCX 26 kb)
