# Supplementary material for: Clinical, physiologic, and radiographic factors contributing to development of hypoxemia in moderate to severe COPD: a cohort study
Source: BMC Pulm Med. 2016 Dec 1;16:169. doi: 10.1186/s12890-016-0331-0 (PMC5131397; doi:10.1186/s12890-016-0331-0)
Supplement: Additional file 1: Table S1. — IRB Approval and Protocol Numbers for COPDGene. (DOCX 26 kb) [file 12890_2016_331_MOESM1_ESM.docx]

**Supplement to:**

**Clinical, Physiologic, and Radiographic Factors Contributing to Development of Hypoxemia in Moderate to Severe COPD**

J. Michael Wells MD^1,2,3^, Raul San Jose Estepar PhD^4^, Merry-Lynn N. McDonald PhD^5^, Surya P. Bhatt MD^1,2^, Alejandro A. Diaz MD^6^, William C. Bailey^1,2^, Francine L. Jacobson MD MPH^4^, Mark T. Dransfield MD^1,2,3^, George R. Washko MD^6^, Barry J. Make MD^7^, Richard Casaburi PhD, MD^8^, Edwin J.R. van Beek MD^9^, Eric A. Hoffman PhD^10^, Frank C. Sciurba MD^11^, James D. Crapo MD^7^, Edwin K. Silverman MD PhD^5^, Craig P. Hersh MD^5^, and the COPDGene Investigators.

^1^Division of Pulmonary, Allergy, and Critical Care and the ^2^Lung Health Center University of Alabama Birmingham, Birmingham, AL, USA; ^3^Birmingham VA Medical Center, Birmingham, AL, USA ; ^4^Department of Radiology, Brigham and Women’s Hospital, Boston, MA, USA; ^5^Channing Division of Network Medicine, Brigham and Women’s Hospital, Harvard Medical School, Boston, MA, USA; ^6^Division of Pulmonary Medicine, Brigham and Women’s Hospital, Boston, MA, USA; ^7^Division of Pulmonary, Critical Care, and Sleep Medicine, National Jewish Health, Denver, CO, USA; ^8^Rehabilitation Clinical Trials Center, Los Angeles Biomedical Research Institute at Harbor UCLA Medical Center, Torrance, CA, USA; ^9^Department of Radiology, University of Edinburgh, Edinburgh, Scotland; ^10^Department of Radiology, University of Iowa, Iowa City, Iowa; ^11^Division of Pulmonary, Allergy, and Critical Care Medicine, University of Pittsburgh, Pittsburgh, PA

Table S1: Page 2

List of COPDGene Investigators: Page 3-4

| Clinical Center | Institution Title | Protocol Number |
| --- | --- | --- |
| National Jewish Health | National Jewish IRB | HS-1883a |
| Brigham and Women’s Hospital | Partners Human Research Committee | 2007-P-000554/2; BWH |
| Baylor College of Medicine | Institutional Review Board for Baylor College of Medicine and Affiliated Hospitals | H-22209 |
| Michael E. DeBakey VAMC | Institutional Review Board for Baylor College of Medicine and Affiliated Hospitals | H-22202 |
| Columbia University Medical Center | Columbia University Medical Center IRB | IRB-AAAC9324 |
| Duke University Medical Center | The Duke University Health System Institutional Review Board for Clinical Investigations (DUHS IRB) | Pro00004464 |
| Johns Hopkins University | Johns Hopkins Medicine Institutional Review Boards (JHM IRB) | NA_00011524 |
| Los Angeles Biomedical Research Institute | The John F. Wolf, MD Human Subjects Committee of Harbor-UCLA Medical Center | 12756-01 |
| Morehouse School of Medicine | Morehouse School of Medicine Institutional Review Board | 07-1029 |
| Temple University | Temple University Office for Human Subjects Protections Institutional Review Board | 11369 |
| University of Alabama at Birmingham | The University of Alabama at Birmingham Institutional Review Board for Human Use | FO70712014 |
| University of California, San Diego | University of California, San Diego Human Research Protections Program | 070876 |
| University of Iowa | The University of Iowa Human Subjects Office | 200710717 |
| Ann Arbor VA | VA Ann Arbor Healthcare System IRB | PCC 2008-110732 |
| University of Minnesota | University of Minnesota Research Subjects’ Protection Programs (RSPP) | 0801M24949 |
| University of Pittsburgh | University of Pittsburgh Institutional Review Board | PRO07120059 |
| University of Texas Health Sciences Center at San Antonio | UT Health Science Center San Antonio Institutional Review Board | HSC20070644H |
| Health Partners Research Foundation | Health Partners Research Foundation Institutional Review Board | 07-127 |
| University of Michigan | Medical School Institutional Review Board (IRBMED) | HUM00014973 |
| Minneapolis VA Medical Center | Minneapolis VAMC IRB | 4128-A |
| Fallon Clinic | IRB/Research Review Committee – St. Vincent Hospital – Fallon Clinic – Fallon Community Health Plan | 1143 |

**Table S1. IRB Approval and Protocol Numbers for COPDGene.**

**COPDGene^®^ Investigators – Core Units**

*Administrative Core*: James Crapo, MD (PI), Edwin Silverman, MD, PhD (PI), Barry Make, MD, Elizabeth Regan, MD, PhD

*Genetic Analysis Core*: Terri Beaty, PhD, Nan Laird, PhD, Christoph Lange, PhD, Michael Cho, MD, Stephanie Santorico, PhD, John Hokanson, MPH, PhD, Dawn DeMeo, MD, MPH, Nadia Hansel, MD, MPH, Craig Hersh, MD, MPH, Peter Castaldi, MD, MSc, Merry-Lynn McDonald, PhD, Emily Wan, MD, Megan Hardin, MD, Jacqueline Hetmanski, MS, Margaret Parker, MS, Marilyn Foreman, MD, Brian Hobbs, MD, Robert Busch, MD, Adel El-Boueiz, MD, Peter Castaldi, MD, Megan Hardin, MD, Dandi Qiao, PhD, Elizabeth Regan, MD, Eitan Halper-Stromberg, Ferdouse Begum, Sungho Won, Sharon Lutz, PhD

*Imaging Core*: David A Lynch, MB, Harvey O Coxson, PhD, MeiLan K Han, MD, MS, MD, Eric A Hoffman, PhD, Stephen Humphries MS, Francine L Jacobson, MD, Philip F Judy, PhD, Ella A Kazerooni, MD, John D Newell, Jr., MD, Elizabeth Regan, MD, James C Ross, PhD, Raul San Jose Estepar, PhD, Berend C Stoel, PhD, Juerg Tschirren, PhD, Eva van Rikxoort, PhD, Bram van Ginneken, PhD, George Washko, MD, Carla G Wilson, MS, Mustafa Al Qaisi, MD, Teresa Gray, Alex Kluiber, Tanya Mann, Jered Sieren, Douglas Stinson, Joyce Schroeder, MD, Edwin Van Beek, MD, PhD

*PFT QA Core, Salt Lake City, UT*: Robert Jensen, PhD

*Data Coordinating Center and Biostatistics*, *National Jewish Health, Denver, CO*: Douglas Everett, PhD, Anna Faino, MS, Matt Strand, PhD, Carla Wilson, MS

*Epidemiology Core*, *University of Colorado Anschutz Medical Campus, Aurora, CO*: John E. Hokanson, MPH, PhD, Gregory Kinney, MPH, PhD, Sharon Lutz, PhD, Kendra Young PhD, Katherine Pratte, MSPH, Lindsey Duca, MS

**COPDGene^®^ Investigators – Clinical Centers**

*Ann Arbor VA:* Jeffrey L. Curtis, MD, Carlos H. Martinez, MD, MPH, Perry G. Pernicano, MD

*Baylor College of Medicine, Houston, TX*: Nicola Hanania, MD, MS, Philip Alapat, MD, Venkata Bandi, MD, Mustafa Atik, MD, Aladin Boriek, PhD, Kalpatha Guntupalli, MD, Elizabeth Guy, MD, Amit Parulekar, MD, Arun Nachiappan, MD

*Brigham and Women’s Hospital, Boston, MA*: Dawn DeMeo, MD, MPH, Craig Hersh, MD, MPH, George Washko, MD, Francine Jacobson, MD, MPH

*Columbia University, New York, NY*: R. Graham Barr, MD, DrPH, Byron Thomashow, MD, John Austin, MD, Belinda D’Souza, MD, Gregory D.N. Pearson, MD, Anna Rozenshtein, MD, MPH, FACR

*Duke University Medical Center, Durham, NC*: Neil MacIntyre, Jr., MD, Lacey Washington, MD, H. Page McAdams, MD

*Health Partners Research Foundation, Minneapolis, MN*: Charlene McEvoy, MD, MPH, Joseph Tashjian, MD

*Johns Hopkins University, Baltimore, MD*: Robert Wise, MD, Nadia Hansel, MD, MPH, Robert Brown, MD, Karen Horton, MD, Nirupama Putcha, MD, MHS,

*Los Angeles Biomedical Research Institute at Harbor UCLA Medical Center, Torrance, CA*: Richard Casaburi, PhD, MD, Alessandra Adami, PhD, Janos Porszasz, MD, PhD, Hans Fischer, MD, PhD, Matthew Budoff, MD, Harry Rossiter, PhD

*Michael E. DeBakey VAMC, Houston*, TX: Amir Sharafkhaneh, MD, PhD, Charlie Lan, DO

*Minneapolis VA:* Christine Wendt, MD, Brian Bell, MD

*Morehouse School of Medicine, Atlanta, GA*: Marilyn Foreman, MD, MS, Gloria Westney, MD, MS, Eugene Berkowitz, MD, PhD

*National Jewish Health, Denver, CO*: Russell Bowler, MD, PhD, David Lynch, MD

*Reliant Medical Group, Worcester, MA*: Richard Rosiello, MD, David Pace, MD

*Temple University, Philadelphia, PA:* Gerard Criner, MD, David Ciccolella, MD, Francis Cordova, MD, Chandra Dass, MD, Gilbert D’Alonzo, DO, Parag Desai, MD, Michael Jacobs, PharmD, Steven Kelsen, MD, PhD, Victor Kim, MD, A. James Mamary, MD, Nathaniel Marchetti, DO, Aditi Satti, MD, Kartik Shenoy, MD, Robert M. Steiner, MD, Alex Swift, MD, Irene Swift, MD, Maria Elena Vega-Sanchez, MD

*University of Alabama, Birmingham, AL:* Mark Dransfield, MD, William Bailey, MD, J. Michael Wells, MD, Surya Bhatt, MD, Hrudaya Nath, MD

*University of California, San Diego, CA*: Joe Ramsdell, MD, Paul Friedman, MD, Xavier Soler, MD, PhD, Andrew Yen, MD

*University of Iowa, Iowa City, IA*: Alejandro Cornellas, MD, John Newell, Jr., MD, Brad Thompson, MD

*University of Michigan, Ann Arbor, MI*: MeiLan Han, MD, Ella Kazerooni, MD, Carlos Martinez, MD

*University of Minnesota, Minneapolis, MN*: Joanne Billings, MD, Tadashi Allen, MD

*University of Pittsburgh, Pittsburgh, PA*: Frank Sciurba, MD, Divay Chandra, MD, MSc, Joel Weissfeld, MD, MPH, Carl Fuhrman, MD, Jessica Bon, MD

*University of Texas Health Science Center at San Antonio, San Antonio, TX*: Antonio Anzueto, MD, Sandra Adams, MD, Diego Maselli-Caceres, MD, Mario E. Ruiz, MD
